# The 6-item InCharge financial distress/financial wellbeing (IFDFW-6) scale: Development and validation in pharmacy and epidemiology research

**DOI:** 10.1016/j.rcsop.2026.100734

**Published:** 2026-03-27

**Authors:** Fouad Sakr, Mariam Dabbous, Jihan Safwan, Mona El Bakri, Mohamad Rahal, Pascale Salameh

**Affiliations:** a School of Pharmacy, Lebanese International University, Beirut, Lebanon; b INSPECT-LB, Institut National de Santé Publique, d'Épidémiologie Clinique et de Toxicologie-Liban, Beirut, Lebanon; c Inserm U1094, IRD UMR270, Univ. Limoges, CHU Limoges, EpiMaCT - Epidemiology of chronic diseases in tropical zone, Institute of Epidemiology and Global Health – Michel Dumas, OmegaHealth, Limoges, France; d Faculty of Pharmacy, Lebanese University, Beirut, Lebanon; e Gilbert and Rose-Marie Chagoury School of Medicine, Lebanese American University, Byblos, Lebanon; f Department of Primary Care and Population Health, University of Nicosia Medical School, Nicosia, Cyprus

**Keywords:** Financial wellbeing, Financial distress, Pharmacy, Epidemiology, IFDFW, Validation

## Abstract

Background: Financial wellbeing and distress have emerged as key social determinants influencing health behaviors, medication adherence, and quality of life. Yet, these constructs remain underexplored in pharmacy and epidemiology research due to the lack of brief, validated, and context-appropriate measurement tools. The widely used InCharge Financial Distress/Financial Wellbeing (IFDFW-8) scale offers strong psychometric evidence but includes items that may not reflect health-related financial strain and may increase respondent burden.

Objective: This study aimed to develop and validate a concise, contextually applicable 6-item version (IFDFW-6) for use in pharmacy and epidemiological research.

Methods: A cross-sectional study was conducted among adults who had recently visited community pharmacies. The questionnaire included validated measures of financial wellbeing (IFDFW-8), medication adherence (SMAS-7), patient experience and satisfaction (MA-PSQ-18), and quality of life (EQ-5D-5L). Exploratory and confirmatory factor analyses (EFA, CFA) evaluated dimensionality; reliability was examined using Cronbach's α and McDonald's ω; criterion validity was tested against the IFDFW-8 using ROC analysis; and measurement invariance was assessed across gender, health status, and healthcare access groups.

Results: Among 501 participants, EFA supported a unidimensional structure explaining 84.3% of variance (factor loadings = 0.878–0.946). CFA confirmed excellent model fit (CFI = 0.997, RMSEA = 0.056, SRMR = 0.008). The IFDFW-6 demonstrated high internal consistency (α = 0.963; ω = 0.963) and strong criterion validity against the parent IFDFW-8 (AUC = 0.993; cutoff = 29.5, sensitivity = 95.8%, specificity = 95.2%). Higher financial wellbeing correlated with better quality of life, greater satisfaction with pharmacy services, and higher medication adherence.

Conclusion: The IFDFW-6 is a reliable and valid instrument that efficiently captures financial wellbeing/distress and has been refined for contextual applicability in pharmacy and epidemiology research, supporting the integration of economic determinants into health-related studies.

## Highlights

• The IFDFW-6 showed a unidimensional structure confirmed by EFA and CFA.
• The scale demonstrated excellent internal consistency (α = 0.963; ω = 0.963).
• Criterion validity was outstanding (AUC = 0.993).
• Measurement invariance was established across gender, health status, and healthcare access.
• Higher financial wellbeing correlated with greater medication adherence, patient satisfaction, and QOL.

## 1 Introduction

Financial wellbeing and financial distress are multidimensional, subjective states describing individuals' perceived capacity to manage day-to-day finances, absorb financial shocks, and achieve future goals.^1^ They encompass emotional, behavioral, and cognitive dimensions of one's financial life and are not fully captured by objective indicators such as income or assets alone.^2^ Although often used interchangeably, financial distress and financial wellbeing represent conceptually distinct but closely related constructs. Financial distress reflects a negatively valenced experiential state characterized by financial stress, worry, and perceived difficulty in meeting current financial obligations, whereas financial wellbeing refers to a broader subjective appraisal encompassing perceived financial control, security, and satisfaction with one's overall financial situation.^1^, ^2^, ^3^ Within health and social science research, these constructs are commonly conceptualized along a single underlying continuum rather than as independent dimensions, such that lower levels of financial wellbeing are expressed through heightened financial distress.^3^, ^4^ The InCharge Financial Distress/Financial Wellbeing (IFDFW) scale operationalizes this continuum by integrating both distress-oriented and wellbeing-oriented items into a unified latent construct, allowing comprehensive assessment of individuals' perceived financial status using a single summary score.^3^ In public health, these constructs align with the social determinants of health framework, in which socioeconomic conditions, such as income, wealth, and material hardship, shape health behaviors and outcomes across the life course.5., 6 In recent years, economic and financial factors have become critical foci in pharmacy, public health, and epidemiology research, given their profound influence on treatment access, medication use, and health equity.7., 8., 9

Financial stress is consistently associated with poorer mental and physical health. Systematic reviews and large observational studies have demonstrated robust links between financial stress or worries and depressive symptoms, psychological distress, and diminished wellbeing.^10^, ^11^ In parallel, cost pressures directly affect medication use: cost sharing and out-of-pocket expenditures are repeatedly linked to lower treatment initiation, reduced adherence, and higher discontinuation, leading to greater healthcare utilization and cost burden.^12^, 13., 14, 15, 16 Cost-related medication nonadherence remains common among adults with chronic conditions and is often under-discussed during clinical encounters, highlighting the need for systematic assessment in research and practice.^17^, ^18^ These dynamics are especially relevant to community pharmacy, where affordability, access, and patient counseling intersect, and are increasingly integrated into models of patient satisfaction, experience, and quality of life (QOL).

Despite the growing recognition of these factors, few validated instruments have been specifically developed to assess financial wellbeing or distress within pharmacy and epidemiology research. Consequently, researchers and practitioners have increasingly relied on the IFDFW scale, as a widely used measure to capture individuals' perceived financial distress and wellbeing.^3^ The IFDFW has shown strong psychometric properties and conceptual clarity across populations; however, certain items may limit its relevance in healthcare contexts. For example, a fixed-amount emergency expense (e.g., “USD 1000”) may be culturally or economically incongruent across countries. In high-income settings, such an amount may represent a manageable short-term expense, whereas in low- and middle-income countries it may exceed several months' income, fundamentally altering respondents' interpretation of the item.^19^ Even within the same instrument, studies conducted across different regions have adopted varying monetary equivalents to approximate local purchasing power, potentially undermining conceptual and metric equivalence across populations.^20^ These discrepancies highlight the challenges of applying fixed monetary thresholds in cross-cultural health research and support the need for context-appropriate item refinement. Furthermore, respondent burden remains a consideration in multi-instrument surveys that also assess medication use, patient satisfaction, and QOL, where concise tools can enhance completion rates and reduce fatigue without compromising reliability.^21^

Developing a shortened, context-appropriate version of the IFDFW could therefore facilitate its integration into pharmacy and epidemiology studies while maintaining psychometric robustness. Best-practice guidelines for scale development emphasize that item reduction should be guided by empirical evidence from factor analysis, reliability, and validity testing, followed by assessment of measurement invariance across key demographic or health subgroups to ensure comparability.^4^ To enhance cross-contextual applicability, contextual and cultural adaptation frameworks recommend refining items to align with the local financial environment, health-system realities, and linguistic nuances, ensuring both conceptual and metric equivalence.^19^, ^20^

With the expanding integration of economic and psychosocial constructs in pharmacy and epidemiology research, measurement precision and validity have become increasingly critical. Instruments used in these fields must demonstrate robust psychometric properties, encompassing reliability, structural validity, and invariance, since practical and policy implications are often derived from such data.^22^, ^23^ Together, these considerations underscore the need for a concise, psychometrically sound, and contextually applicable tool to assess financial wellbeing and distress within health research frameworks.

Accordingly, this study aimed to develop and psychometrically validate a shortened version of the IFDFW (the 6-item IFDFW-6) for use in pharmacy and epidemiology research. Specifically, the study evaluated dimensionality through exploratory and confirmatory factor analyses, assessed internal consistency and measurement invariance across health-related subgroups, and examined criterion validity against the original 8-item scale (IFDFW-8). Concurrent validity was further tested against constructs central to pharmacy and epidemiology, including medication adherence, patient experience and satisfaction, and health-related QOL, to provide a brief yet rigorous instrument for investigating the financial determinants of public health.

## 2 Methods

### 2.1 Development and content validity of the 6-item IFDFW scale

The development of the IFDFW-6 scale was undertaken through two complementary approaches, data-driven and theoretical refinement of the original 8-item IFDFW scale. Initially, an Exploratory Factor Analysis (EFA) was conducted to examine the latent structure and factor loadings of the IFDFW-8, while internal consistency reliability was assessed using Cronbach's α following sequential item deletion. Items 5 and 6 demonstrated the lowest factor loadings and communalities in the EFA, and their content raised concerns regarding contextual relevance for health research settings. Changes in Cronbach's α following item removal were examined as a supplementary indicator of internal consistency; however, item-retention decisions were guided primarily by factor analytic results and theoretical considerations rather than by α optimization alone, given the known tendency of α to increase with item reduction. Details of these preliminary analyses are provided in Appendix 1.

Subsequently, a panel of six experts, all study authors, comprising five pharmacists (four pharmacoepidemiologists, three pharmacotherapy specialists, and one clinical pharmacist) and one pharmacologist engaged in two rounds of a modified Delphi technique to refine the scale based on the empirical findings. Each item was independently rated for retention or removal on a binary scale (retain = 1; remove = 0), with ≥80% consensus required for exclusion. The panel also provided qualitative feedback on item clarity and contextual relevance. Both rounds were conducted anonymously to minimize bias and ensure independent judgment.

Item 5 (“How confident are you that you could find the money to pay for a financial emergency that costs about 1000 USD?”) was removed due to its limited cross-contextual applicability, as the reference currency varied substantially across studies and settings, particularly within the Lebanese context, where arbitrary equivalents (e.g., USD 250 or USD 500) were sometimes adopted. Item 6 (“How often does this happen to you? You want to go out to eat, go to a movie, or do something else and don't go because you can't afford to?”) was also excluded, as it was deemed conceptually irrelevant to pharmacy, epidemiology, and public health research contexts. Since both theoretical and data-driven approaches converged on the same conclusions, the refined IFDFW retained six items (1, 2, 3, 4, 7, 8) of the original scale. A subsequent EFA was then performed to confirm the latent structure of the shortened IFDFW-6 version.

Conceptually, the retained IFDFW-6 items reflect core content facets of financial wellbeing/distress that are directly relevant to health research contexts, including perceived financial control, financial stress, worry about meeting essential expenses, and overall satisfaction with one's financial situation. In contrast, the excluded items primarily assessed context-dependent behaviors or fixed monetary thresholds, such as the ability to cover a specific emergency expense or discretionary leisure activities. While these items may be informative in consumer or financial-planning research, they are less directly aligned with health-related financial strain and are more sensitive to contextual, economic, and income-distribution differences. The retained items therefore preserve the conceptual breadth of the IFDFW continuum while enhancing contextual relevance and interpretability for pharmacy and epidemiology research.

Although the Delphi panel consisted exclusively of study authors, several measures were implemented to minimize potential bias and preserve independence in decision-making. Both Delphi rounds were conducted anonymously, preventing direct interaction or dominance by any individual panel member. Item-retention decisions were primarily guided by empirical evidence derived from EFA and reliability testing, with expert judgment limited to evaluating contextual relevance and conceptual clarity. In addition, panel members represented complementary disciplinary backgrounds, and none were involved in modifying or rewriting items during data collection. Importantly, the data-driven and theoretical approaches converged on identical item-removal decisions, supporting the robustness of the final item selection and reducing the likelihood that subjective preferences influenced the scale refinement process.

### 2.2 Study design

A cross-sectional survey was carried out in Lebanon from February to May 2025. Eligible participants were adults (≥18 years) who had received services from a community pharmacist or pharmacy within the preceding six months. Recruitment followed a snowball sampling strategy, with the questionnaire administered electronically via Google Forms and circulated on major social media platforms (WhatsApp, Facebook, Instagram, and LinkedIn) to capture participants from all Lebanese districts.

The survey's introductory section outlined the study aims, estimated completion time, and voluntary nature of participation, emphasizing that respondents could discontinue at any stage. A pilot test involving ten participants was conducted to ensure item clarity and face validity, after which minor modifications were applied. Pilot data were excluded from the final analysis.

### 2.3 Instrument and variables

The Arabic-language questionnaire comprised five main sections. All included scales were already available in Arabic; therefore, no further translation or linguistic adaptation was required. The first section gathered sociodemographic information such as age, gender, and indicators of financial wellbeing. Financial wellbeing/distress was measured using the original 8-item InCharge Financial Distress/Financial Wellbeing (IFDFW) scale, rated from 1 (lowest) to 10 (highest), with higher scores reflecting greater financial wellbeing.^3^ In the present study, internal consistency reliability was excellent, Cronbach's α = 0.960.

The second section collected clinical information, including current health status, chronic conditions, and access to healthcare services. Medication adherence was assessed using the Short Medication Adherence Scale (SMAS-7), a validated 7-item instrument scored on a 4-point Likert scale (1 = low adherence to 4 = high adherence), where higher scores denote better adherence.^24^ Reliability in this study was strong (α = 0.889).

The third section focused on patients' experiential characteristics related to community pharmacy visits, assessed through the Patient-Pharmacist Relationship Measurement Tool, previously validated in Lebanon.^25^ This tool comprises three subscales: the Patient Expectation Index (11 items; α = 0.745), the Barriers to Communication Index (7 items; α = 0.905), and the Patient Perception Index (14 items; α = 0.905). Higher scores correspond to greater expectations, more perceived barriers, or more favorable perceptions, respectively.

The fourth section evaluated satisfaction with pharmacy services using the modified 18-item Patient Satisfaction Questionnaire Short Form (MA-PSQ-18).^26^ The instrument captures satisfaction across multiple domains, including general satisfaction, technical quality, interpersonal manner, time spent with the pharmacist, and accessibility/convenience. Subscale and total scores were computed by summing item responses, with higher scores reflecting greater satisfaction (α = 0.952).

Finally, the fifth section assessed health-related QOL using the EQ-5D-5L, which measures five dimensions: mobility, self-care, usual activities, pain/discomfort, and anxiety/depression, along with the EQ Visual Analogue Scale (EQ-VAS) ranging from 0 (worst imaginable health) to 100 (best imaginable health).^27^

### 2.4 Ethical aspects

The study protocol received approval from the Ethics and Research Committee of the School of Pharmacy at the Lebanese International University (Approval No. 2025ERC-009-LIUSOP). Participation was entirely voluntary, and informed consent was obtained electronically from all respondents prior to data collection. The research was conducted in accordance with the ethical principles outlined in the Declaration of Helsinki, with strict measures implemented to maintain participant confidentiality and uphold research integrity.

### 2.5 Sample size requirements

For the validation process, a participant-to-item ratio of 10:1^4^ indicated that at least 60 participants were needed for the six IFDFW-6 items, a number that was doubled to 120 to allow validation across two independent subsamples. The sample size was further determined using G*Power version 3.1.9.7 (Heinrich Heine University, Düsseldorf, Germany) to evaluate concurrent validity and correlations with related constructs. Assuming a two-tailed Pearson correlation with a small expected effect size (*r* = 0.20), a significance level of α = 0.05, and a desired power of 0.80, the analysis yielded a minimum required sample of 193 participants. Accordingly, a sample of 193 respondents was deemed sufficient to ensure a 95% confidence level and 80% statistical power for scale validation and association analyses.

### 2.6 Data analysis

Data analyses were performed using R version 4.5.1 (R Foundation for Statistical Computing, Vienna, Austria) within RStudio version 2025.09.1 + 401 (Cucumberleaf Sunflower, Posit Software, PBC). Descriptive statistics were used to summarize participants' sociodemographic, clinical, and experiential characteristics. Continuous variables were presented as means ±standard deviations (SD), while categorical variables were summarized as frequencies and percentages.

The dataset was randomly split into two equal subsets. The first subset was used to conduct an initial EFA to guide item refinement and reduction of the IFDFW scale, as described in the development phase. The second subset underwent a follow-up EFA to reconfirm the factor structure of the retained items. EFAs were carried out using the *psych* package with principal component extraction, following verification of sampling adequacy through the Kaiser-Meyer-Olkin (KMO) measure and Bartlett's test of sphericity. Factors with eigenvalues >1 were retained. Exploratory analyses were conducted using principal component analysis (PCA) as a data-reduction approach to support item refinement and scale shortening. PCA was selected to identify items with high shared variance and potential redundancy in the context of developing a brief instrument.

Subsequently, a confirmatory factor analysis (CFA) was conducted on the second subset using the *lavaan* package with maximum likelihood estimation to validate the dimensional structure derived from EFA. Model fit was evaluated using multiple indices: χ^2^/df, Comparative Fit Index (CFI), Tucker-Lewis Index (TLI), Root Mean Square Error of Approximation (RMSEA), and Standardized Root Mean Square Residual (SRMR). Good model fit was defined as χ^2^/df < 3, CFI and TLI ≥ 0.95, RMSEA ≤0.08, and SRMR ≤0.08.^28^ Standardized factor loadings and the path diagram were generated using IBM SPSS Amos version 24.

All subsequent analyses were conducted on the entire sample. Measurement invariance of the IFDFW-6 was evaluated via multigroup CFA across gender, health status, and access to healthcare. Configural, metric, and scalar invariance were assessed sequentially, with invariance accepted if ΔCFI ≤0.010, ΔRMSEA ≤0.015, and ΔSRMR ≤0.010.^22^

Internal consistency reliability was examined using Cronbach's α (*psych* package) and McDonald's ω (*semTools*), along with item-total Pearson correlations. Agreement with the parent instrument was assessed via ROC curve analysis using the *pROC* and *ggplot2* packages, with the original IFDFW-8 serving as the reference measure. Given that the IFDFW-6 was derived from the IFDFW-8, this analysis evaluates concordance with the parent scale rather than validation against an independent external criterion. A total score of 40 on the IFDFW-8 was applied as the cut-off between financial wellbeing and financial distress,^3^ and the optimal IFDFW-6 threshold was determined using Youden's J index to balance sensitivity and specificity.

Concurrent validity and associations with relevant constructs were evaluated through Pearson's correlation coefficients (r) between the IFDFW-6 total score and variables including age, number of comorbidities, number of routine medications, SMAS-7 score, MA-PSQ-18 score, Patient Expectation Index, Barriers to Communication with Pharmacist score, Patient Perception Index, and EQ-VAS score. Statistical significance was set at *P* < 0.05 for all analyses.

## 3 Results

### 3.1 Sociodemographic, clinical, and experiential characteristics

Table 1 summarizes the sociodemographic, clinical, and experiential characteristics of the study participants. The sample comprised 501 individuals, of whom 67.27% were female and 32.73% were male. Most participants reported no chronic illness (74.05%), and the majority indicated easy access to healthcare (79.84%). The mean age was 34.66 ± 15.17 years, with participants reporting an average of 1.33 ± 1.56 comorbidities and 1.04 ± 1.66 chronic daily medications. Regarding experiential measures, the mean Patient Expectation Index score was 20.09 ± 2.01, and the mean Barriers for Communication with Pharmacist score was 9.69 ± 2.70. Participants also reported a mean Patient Perception Index score of 35.22 ± 6.51, a mean SMAS-7 score of 18.64 ± 5.78, and a mean EQ-VAS score of 76.16 ± 20.82.

**Table 1:** Sociodemographic, clinical, and experiential characteristics of participants.

**Variable**	**Frequency**	**%**
*Gender*		
Male	164	32.73
Female	337	67.27
*Current health status*		
No chronic illness	371	74.05
Chronic illness	130	25.95
*Easy access to healthcare*		
No	101	20.16
Yes	400	79.84
**Variable**	**Mean**	**SD**
*Age*	34.66	15.17
*Total number of comorbidities*	1.33	1.56
*Number of chronic daily medications*	1.04	1.66
*Patient Expectation Index score*	20.09	2.01
*Barriers for Communication with Pharmacist score*	9.69	2.70
*Patient Perception Index score*	35.22	6.51
*SMAS-7 score*	18.64	5.78
*EQ-VAS score*	76.16	20.82

SD: standard deviation; SMAS-7: 7-item Short Medication Adherence Scale; EQ-VAS: EuroQol Visual Analogue Scale.

### 3.2 Exploratory factor analysis

A second EFA was conducted on Sample 1 (*N* = 250) after item refinement to assess the factor structure of the finalized IFDFW-6. A one-factor solution was retained, with all six items loading strongly on the single component (factor loadings ranging from 0.878 to 0.946) and explaining 84.3% of the total variance. Sampling adequacy was excellent (KMO = 0.901), and Bartlett's test of sphericity was significant (*P* < 0.001), confirming the suitability of the data for factor analysis. The results of the principal component analysis of the IFDFW-6 are presented in Table 2.

**Table 2:** Principal component analysis of the IFDFW-6 in Sample 1.

**IFDFW-8 item #**	**IFDFW-6 item #**	**IFDFW item**	**Factor loading**	**h**^**2**^
3	1	How do you feel about your current financial situation?	0.946	0.894
4	2	How often do you worry about being able to meet normal monthly living expenses?	0.933	0.870
8	3	How stressed do you feel about your personal finances in general?	0.929	0.863
2	4	How satisfied you are with your present financial situation?	0.915	0.837
1	5	What do you feel is the level of your financial stress today?	0.908	0.825
7	6	How frequently do you find yourself just getting by financially and living paycheck to paycheck?	0.878	0.771
		*% of variance explained*	84.32%	

Kaiser-Meyer-Olkin (KMO) Measure of Sampling Adequacy = 0.901.

Bartlett's Test of Sphericity: *P* < 0.001.

h^2^: communalities.

### 3.3 Confirmatory factor analysis

**Fig. 1:**
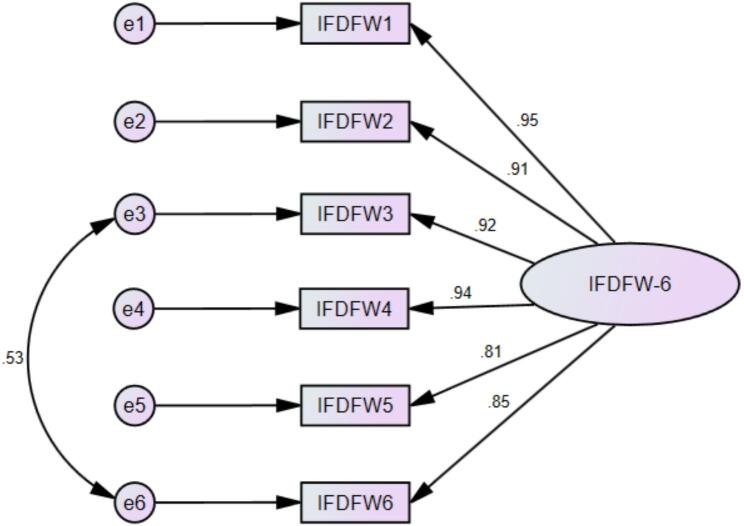
Standardized loadings for the IFDFW-6 items derived from the confirmatory factor analysis (CFA) in Sample 2. e = error term.

To validate the structure identified in the EFA, a CFA was conducted on Sample 2 (*N* = 251). The initial unmodified model showed suboptimal fit (χ^2^/df = 78.013/9 = 8.668; CFI = 0.963; TLI = 0.938; RMSEA = 0.175 [90% CI = 0.141–0.212, P < 0.001]; SRMR = 0.020). Guided by theoretically justifiable modification indices, a residual covariance was introduced between two items referencing routine financial strain. This modification accounted for statistically significant shared item-specific variance while preserving the unidimensional latent structure of the scale. Without this modification, optimal overall model fit was not achieved. The adjusted model demonstrated a substantial improvement and yielded excellent fit indices (χ^2^/df = 14.293/8 = 1.787; CFI = 0.997; TLI = 0.994; RMSEA = 0.056 [90% CI = 0.001–0.102, *P* = 0.363]; SRMR = 0.008). All standardized factor loadings were strong (Fig. 1).

### 3.4 CFA multi-group measurement invariance

Multi-group CFA demonstrated configural, metric, and scalar invariance of the IFDFW-6 across gender, health status, and access to healthcare groups (Table 3). All models showed excellent fit (CFI = 0.999; SRMR ≤0.024). Although RMSEA values were slightly inflated in some configural and metric models (up to 0.12), this inflation is common in models with small degrees of freedom, where RMSEA tends to overestimate lack of fit despite adequate model performance.^28^ Differences in fit indices across levels (ΔCFI <0.001; ΔSRMR ≤0.006) were well below recommended thresholds, supporting measurement equivalence of the scale across groups.

**Table 3:** IFDFW-6 measurement invariance testing across gender, health status, and healthcare access groups.

**Model**	**CFI**	**RMSEA**	**SRMR**	**Model comparison**	**ΔCFI**	**ΔRMSEA**	**ΔSRMR**
**Model 1: across gender (male vs. female)**							
Configural	0.999	0.100	0.018				
Metric	0.999	0.124	0.024	Configural vs metric	< 0.001	0.024	0.006
Scalar	0.999	0.042	0.018	Metric vs scalar	< 0.001	0.082	0.006
**Model 2: across current health status (chronic illness vs. no illness)**							
Configural	0.999	0.105	0.018				
Metric	0.999	0.107	0.020	Configural vs metric	< 0.001	0.002	0.002
Scalar	0.999	0.064	0.018	Metric vs scalar	< 0.001	0.043	0.002
**Model 3: across easy access to healthcare (yes vs. no)**							
Configural	0.999	0.071	0.016				
Metric	0.999	0.105	0.022	Configural vs metric	< 0.001	0.034	0.006
Scalar	0.999	0.018	0.017	Metric vs scalar	< 0.001	0.087	0.005

CFI: Comparative Fit Index; RMSEA: Root Mean Square Error of Approximation; SRMR: Standardized Root Mean Square Residual.

### 3.5 Reliability and internal consistency

**Fig. 2:**
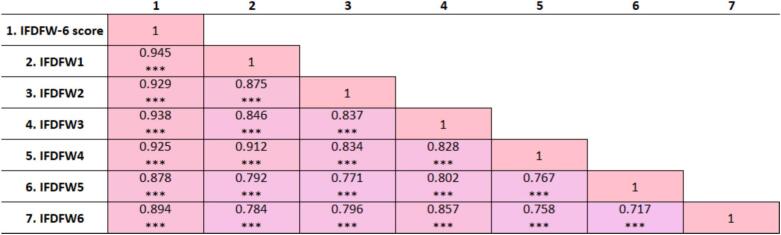
Pearson correlation matrix for the IFDFW-6 items and total score. IFDFW1 to IFDFW6 (IFDFW-6 scale item numbers). ***P < 0.001.

The IFDFW-6 demonstrated excellent reliability, with Cronbach's α = 0.963 and McDonald's ω = 0.963. All correlations were positive and statistically significant (*P* < 0.001). The total IFDFW-6 score showed strong associations with each item (*r* = 0.878–0.945), and inter-item correlations were similarly high (*r* = 0.717–0.912), reflecting substantial internal coherence among the six items and supporting the unidimensional structure of the scale (Fig. 2).

### 3.6 Agreement with the parent instrument (ROC analysis)

**Fig. 3:**
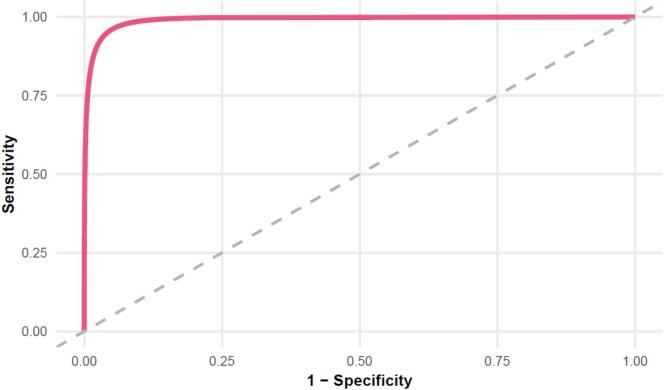
ROC curve of the IFDFW-6. Participants classified as having acceptable financial wellbeing on the IFDFW-8 were analyzed. The area under the curve was 0.993 (95% CI = 0.988–0.997, P < 0.001). At a cutoff value of 29.50, sensitivity was 95.8% and specificity was 95.2%.

Agreement between the IFDFW-6 and the original IFDFW-8 was assessed using ROC curve analysis comparing participants classified as financially well versus financially distressed according to the IFDFW-8. Given that the IFDFW-6 was derived from the IFDFW-8, this analysis reflects internal classification concordance rather than independent criterion validity. The IFDFW-6 yielded an optimal cutoff value of 29.50, derived from statistical agreement with the parent instrument, with a sensitivity of 95.8% and a specificity of 95.2%. The area under the curve (AUC) was 0.993 (95% CI = 0.988–0.997, *P* < 0.001). This threshold should be considered provisional and not yet clinically validated. The ROC curve for the IFDFW-6 is illustrated in Fig. 3.

### 3.7 Concurrent validity and associations with pharmacy- and epidemiology-related constructs

The IFDFW-6 had a mean score of 35.04 ± 15.31 (range: 6.00–60.00). The IFDFW-6 score demonstrated several significant correlations with theoretically related constructs within the pharmacy and epidemiology contexts. Higher financial wellbeing was moderately associated with better QOL on the EQ-VAS (*r* = 0.334, *P* < 0.001) and with greater satisfaction with community pharmacy services measured by the MA-PSQ-18 (*r* = 0.173, P < 0.001). Positive correlations were also found with the Patient Perception Index (*r* = 0.120, *P* = 0.007) and medication adherence assessed by the SMAS-7 (*r* = 0.119, *P* = 0.008). The Patient Expectation Index showed a borderline significant correlation with the IFDFW-6 (*r* = 0.086, *P* = 0.055). In contrast, a higher number of comorbidities was significantly associated with lower financial wellbeing (*r* = −0.135, *P* = 0.003), whereas correlations with barriers to communication with pharmacists (*r* = −0.061, *P* = 0.173), age (*r* = 0.007, *P* = 0.875), and number of routine daily medications (*r* = 0.002, *P* = 0.970) were not significant.

## 4 Discussion

This study developed and psychometrically validated a concise six-item version of the InCharge Financial Distress/Financial Wellbeing scale (IFDFW-6) for use in pharmacy and epidemiology research. The IFDFW-6 demonstrated a robust unidimensional structure, excellent internal consistency, strong criterion validity, and measurement invariance across gender, health status, and access to healthcare. These findings confirm that financial wellbeing and distress can be reliably captured with fewer items while maintaining conceptual integrity and measurement precision.

The construct validity analysis revealed that the IFDFW-6 retained the theoretical coherence of the original eight-item scale, with all items loading strongly on a single latent dimension. This supports previous evidence that financial wellbeing/distress is a unified construct encompassing satisfaction, stress, and perceived control over personal finances rather than distinct subdomains.^29^ The IFDFW-6 captures a unidimensional subjective continuum of financial wellbeing and distress, emphasizing perceived financial strain, control, and overall appraisal rather than objective financial indicators or behavioral domains. Exploratory analyses supported a single latent factor, consistent with the theoretical positioning of the IFDFW as a unified construct. Given the limited number of items, alternative structural representations (e.g., correlated two-factor or bifactor models) would involve few indicators per factor and may yield unstable estimates. Future research in larger and more heterogeneous samples may formally compare alternative models to further evaluate the robustness of this continuum framework. The factor loadings are comparable to those reported for the original IFDFW-8,^3^ and align with studies validating similar instruments, such as the CFPB Financial Well-Being Scale.^30^ Although the IFDFW captures multiple content facets of financial wellbeing/distress (e.g., stress, satisfaction, perceived control), these facets are theorized to reflect a single underlying construct rather than separable dimensions, supporting the unidimensional structure observed in both the original and shortened versions of the scale. The high reliability indices further underscore the internal homogeneity of the six retained items, exceeding the psychometric thresholds recommended for instruments used in clinical and epidemiological research.^31^

Together, these psychometric findings provide a stable foundation for examining how financial wellbeing/distress interfaces with health-related constructs. The CFA yielded excellent fit indices after introducing a residual covariance between two items referencing routine financial strain. Although both items relate to day-to-day financial pressure, one focuses on anticipatory worry about meeting monthly expenses, whereas the other reflects perceived financial sufficiency in terms of “just getting by” or living paycheck to paycheck. Their shared residual variance likely reflects their common reference to ongoing financial obligations rather than redundancy in content, a pattern frequently observed in brief scales measuring closely related experiential states.^32^ Importantly, the modification preserved strong factor loadings and did not alter the substantive interpretation of the latent construct. The demonstration of configural, metric, and scalar invariance across gender, health status, and healthcare access provides strong evidence of measurement equivalence, an aspect rarely examined in prior financial wellbeing/distress research. Such invariance ensures that observed differences in IFDFW-6 scores reflect genuine variations in financial wellbeing/distress rather than measurement bias.^22^

The very high internal consistency coefficients (α and ω > 0.95) and inter-item correlations observed in the IFDFW-6 reflect strong conceptual homogeneity among items capturing perceived financial strain, stress, and control. While such homogeneity supports unidimensionality and reliability, very high reliability may also indicate reduced construct breadth due to closely related item content. This reflects a common trade-off in brief instruments, where efficiency and precision are balanced against broader construct coverage. The IFDFW-6 was intentionally optimized for parsimony and contextual relevance in pharmacy and epidemiology research; however, future studies may explore whether alternative item configurations could further expand construct representation while maintaining acceptable reliability.

Criterion validity was excellent, indicating that the IFDFW-6 effectively distinguishes between financially-distressed and financially-well individuals based on the original IFDFW-8 threshold. This discriminative capacity surpasses what is typically observed for psychosocial instruments, where AUCs between 0.80 and 0.90 are generally considered strong.^33^ The scale's ability to capture gradations of financial strain highlights its sensitivity and potential utility in both research and practice. The ROC analysis demonstrated a very high level of classification accuracy, reflecting strong concordance with the parent IFDFW-8. However, because the IFDFW-6 was directly derived from the original scale, the observed agreement is partly attributable to structural overlap between the two instruments. Accordingly, these findings should be interpreted as evidence of internal classification concordance rather than independent external criterion validity. In addition, the proposed cutoff score was statistically derived from agreement with the parent instrument and was not established using anchor-based or distribution-based methods; therefore, it should be considered provisional and not yet clinically validated. Future studies should evaluate the IFDFW-6 against external financial hardship indicators, objective socioeconomic measures, and longitudinal anchors to establish broader external validity and clinically meaningful interpretability.

The concurrent validity findings further emphasize the IFDFW-6's relevance in pharmacy and epidemiology contexts. Higher financial wellbeing was associated with better health-related QOL, greater satisfaction with pharmacy services, more positive patient perceptions, and higher medication adherence. These relationships mirror theoretical and empirical evidence that financial wellbeing shapes individuals' capacity and motivation to engage in healthy behaviors and maintain treatment continuity.34., 35.

Contextual application is essential in measuring financial wellbeing/distress, as perceptions of financial security and stress are shaped by local economic and social contexts.^30^, ^32^ In refining the IFDFW-6, item reduction was guided by both statistical performance and principles of conceptual equivalence, prioritizing universally interpretable experiences of financial strain and control over context-specific monetary thresholds or behaviors.^32^ The removal of fixed-amount emergency-expense and discretionary spending items was therefore intended to enhance cross-contextual applicability. Importantly, the demonstration of configural, metric, and scalar invariance across gender, health status, and healthcare access groups provides empirical support for the robustness of the IFDFW-6 within the study context, while further cross-national validation remains warranted.

The association between financial wellbeing and QOL is consistent with cross-national studies showing that individuals experiencing less financial stress report higher self-rated health and wellbeing.36., 37 Financial security reduces psychological distress, enhances autonomy, and allows allocation of resources to preventive or maintenance care, explaining this relationship.^38^ In Lebanon and other middle-income countries facing economic volatility, perceived financial control has been strongly linked to mental health and life satisfaction,^39^ reinforcing the contextual relevance of these findings.

The observed association between higher financial wellbeing and greater satisfaction with community pharmacy services aligns with prior research showing that financially stable patients report higher satisfaction, likely because they encounter fewer affordability barriers and perceive service interactions more positively.^40^ Similar findings were reported in the literature: financial strain was linked to lower satisfaction with care, and poorer provider communication corresponded to lower perceived quality.^41^ Within pharmacy practice, this association suggests that financial strain may indirectly affect patients' trust, engagement, and receptiveness to pharmacist counseling.

The positive link between financial wellbeing and medication adherence corroborates previous evidence connecting cost-related burden to poor adherence.^42^ Economic hardship constrains patients' ability to purchase prescribed medications, leading to dose rationing or discontinuation. Conversely, better financial wellbeing enhances adherence by supporting affordability, psychological stability, and proactive health behavior.^43^ Although the association was modest, its significance remains meaningful in behavioral health research, where adherence is influenced by numerous interacting factors.

The significant relationship between financial wellbeing and patient perceptions of the pharmacist further suggests a psychological spillover effect: reduced financial stress enhances cognitive bandwidth, optimism, and satisfaction with interpersonal experiences.^44^ Financial distress, in contrast, may exacerbate frustration and perceived barriers to healthcare interaction.

Some correlations, however, were non-significant, particularly those with age, number of medications, and barriers to communication. The lack of association with age and polypharmacy indicates that financial wellbeing is not merely a reflection of biological or treatment burden but a distinct psychosocial construct shaped by perceived control rather than absolute need. Similar findings were observed in U.S. and Korean cohorts where financial stress did not vary significantly with age after accounting for income and coping strategies.^45^, ^46^ The absence of association with communication barriers likely reflects that communication challenges are more strongly influenced by system-level or provider-level factors, such as time constraints, language, or health literacy, than by financial circumstances.^47^ These null associations are informative, suggesting that financial wellbeing/distress primarily influences motivational and affective aspects of healthcare engagement rather than structural or demographic characteristics.

Collectively, these results demonstrate that the IFDFW-6 captures a construct that is both psychologically and behaviorally consequential. It links financial wellbeing with adherence, satisfaction, and perceived QOL, key outcomes in pharmacy and epidemiological research, thereby filling an important gap by providing a concise, validated measure of a determinant that is widely recognized but seldom quantified in health studies.

### 4.1 Implications for research and practice

The IFDFW-6 can be easily integrated into community pharmacy and population-based surveys to identify individuals experiencing financial strain that may compromise medication use, satisfaction, or wellbeing. In practice, the IFDFW-6 could be incorporated into routine community pharmacy encounters as a brief screening tool to identify patients experiencing financial strain that may compromise medication adherence or treatment continuity. Pharmacists could use IFDFW-6 scores to guide tailored counseling, discuss cost-related concerns, and refer patients to medication-assistance programs, generic alternatives, or insurance-related support services when available. At the health-system level, the IFDFW-6 could be embedded within electronic health records or population health surveys to monitor financial vulnerability, inform resource allocation, and evaluate the impact of policies or interventions aimed at reducing economic barriers to care. Routine application of such tools can help pharmacists tailor counseling, direct patients toward financial-assistance or medication-support programs, and inform policy efforts aimed at reducing economic barriers to care. However, given the relatively young and digitally connected profile of the present sample, further validation in older, clinically complex, and socioeconomically vulnerable populations is recommended before broad routine implementation in diverse pharmacy settings. For researchers, incorporating financial wellbeing into health-behavior models may clarify pathways linking economic hardship with adherence, treatment persistence, and patient-reported outcomes. Given its brevity and proven invariance, the IFDFW-6 is particularly suitable for large-scale studies that require concise yet psychometrically sound measures.

### 4.2 Strengths and limitations

The study's strengths include its rigorous, theory-informed item reduction process that combined empirical evidence from exploratory and confirmatory factor analyses with theoretical consensus, adequate statistical power, and comprehensive validation encompassing reliability, criterion validity, and measurement invariance. The inclusion of multiple health-related constructs allowed a multidimensional examination of concurrent validity within a real-world pharmacy context. Limitations include the cross-sectional design, which restricts causal inference and precludes conclusions about the temporal or directional relationships between financial wellbeing/distress and related outcomes. In addition, test-retest reliability was not evaluated in the present study. As temporal stability is a core psychometric property under contemporary validation standards, future research should assess intraclass correlation coefficients to further establish the stability of the IFDFW-6 over time. Standard Error of Measurement and Smallest Detectable Change were not estimated, and responsiveness or longitudinal sensitivity of the IFDFW-6 has not yet been established, which may limit its interpretability for monitoring change in clinical or pharmacy practice settings. A methodological consideration concerns the use of PCA with the Kaiser criterion during the exploratory phase. PCA was applied as a data-reduction technique to support item refinement and scale shortening. Although PCA and eigenvalue >1 criteria are widely used, they do not model latent variance in the manner of common factor extraction methods, and the Kaiser criterion may overestimate dimensionality in some contexts. In the present study, however, a clear single component emerged with strong loadings and substantial explained variance, and the structure was subsequently confirmed through CFA within a latent variable framework. Future research may strengthen structural validity evidence by applying common factor extraction methods in combination with parallel analysis. The online snowball sampling strategy resulted in a sample that was relatively young, predominantly female, and largely free of chronic illness, which may limit generalizability to older or more medically vulnerable populations for whom financial distress may be more salient. This sampling profile may also contribute to range restriction in financial distress levels and to inflated psychometric estimates, including internal consistency coefficients and model fit indices, thereby potentially influencing observed structural performance. Self-reported data may be subject to recall or social-desirability bias. While the IFDFW-6 demonstrated robust psychometric properties in this study, replication in more diverse clinical and sociodemographic populations, as well as in longitudinal designs, is warranted to confirm generalizability, predictive validity, and responsiveness to change.

## 5 Conclusion

The IFDFW-6 exhibited strong psychometric robustness and meaningful concurrent validity with key health outcomes, confirming its suitability as a concise and contextually applicable measure of financial wellbeing and distress in pharmacy and epidemiology research. Beyond its methodological contribution, the IFDFW-6 offers practical value for identifying populations at financial risk and informing interventions that address the economic determinants of medication adherence, patient satisfaction, and overall QOL. Future studies should assess its predictive validity and responsiveness to interventions targeting financial counseling or cost-related barriers, further establishing its role in promoting both economic and health resilience.

## CRediT authorship contribution statement

**Fouad Sakr:** Writing – review & editing, Writing – original draft, Visualization, Validation, Supervision, Software, Resources, Project administration, Methodology, Investigation, Formal analysis, Data curation, Conceptualization. **Mariam Dabbous:** Writing – review & editing, Writing – original draft. **Jihan Safwan:** Writing – review & editing, Conceptualization. **Mona El Bakri:** Writing – review & editing, Conceptualization. **Mohamad Rahal:** Writing – review & editing. **Pascale Salameh:** Writing – review & editing.

## Consent for publication

Not applicable.

## Funding

This research did not receive any specific grant from funding agencies in the public, commercial, or not-for-profit sectors.

## Declaration of competing interest

The authors have nothing to declare.

## Appendix A Supplementary data

Supplementary material Image 1
