# Supplementary material for: The 6-item InCharge financial distress/financial wellbeing (IFDFW-6) scale: Development and validation in pharmacy and epidemiology research
Source: Explor Res Clin Soc Pharm. 2026 Mar 27;22:100734. doi: 10.1016/j.rcsop.2026.100734 (PMC13089190; doi:10.1016/j.rcsop.2026.100734)
Supplement: Supplementary file 1 — Supplementary material [file mmc1.pdf]

Exploratory Factor Analysis and Reliability Statistics for the Original 8-Item InCharge Financial Distress/Financial Wellbeing (IFDFW-8) Scale

IFDFW item #	IFDFW item	Factor loading	Cronbach's α if item is removed
4	How often do you worry about being able to meet normal monthly living expenses?	0.930	0.951
3	How do you feel about your current financial situation?	0.927	0.952
8	How stressed do you feel about your personal finances in general?	0.922	0.952
7	How frequently do you find yourself just getting by financially and living paycheck to paycheck?	0.897	0.953
2	How satisfied you are with your present financial situation?	0.895	0.954
1	What do you feel is the level of your financial stress today?	0.891	0.955
5	How confident are you that you could find the money to pay for a financial emergency that costs about 500 USD?	0.821	0.959
6	How often does this happen to you? You want to go out to eat, go to a movie or do something else and don't go because you can't afford to?	0.802	0.960
	% of variance explained	0.786	

Kaiser-Meyer-Olkin Measure (KMO) of Sampling Adequacy = 0.927

Bartlett's Test of Sphericity ($P < 0.001$)

Cronbach's α with all items = 0.960
